# Food Microbiology: The Past and the New Challenges for the Next 10 Years

**DOI:** 10.3389/fmicb.2020.00237

**Published:** 2020-02-21

**Authors:** Giovanna Suzzi, Aldo Corsetti

**Affiliations:** Faculty of BioScience and Technology for Food, Agriculture and Environment, University of Teramo, Teramo, Italy

**Keywords:** foods analysis, food–borne pathogens, food safety and quality, bioactive compounds, probiotics

Food microbiology papers published during the past decade have been characterized by multidisciplinary interests that have confirmed the increasing amount of evidence that has implicated microorganisms in different areas, including food technology, food safety and hygiene, food poisoning, food genomics, and, more generally, food omics, functional foods, and probiotics, besides emerging methodologies that have been applied to food analyses. Probiotics research and innovation in functional food production deserves particular attention. Many articles have focused on the survival of potential probiotic bacteria in the gastro-intestinal tract (GIT), the microbial adhesive capacity and colonization of the gut, the safety status of probiotic strains, as well as gut microbiome homeostasis maintenance by competitively inhibiting the growth of pathogens or producing antimicrobial compounds. However, new probiotic strains are (or will be) screened for natural bioactive substances, immunomodulation capacity, as well as anticancer and other health benefits. Fifteen Research Topics (RTs) on these important subjects have been submitted to the Food Microbiology section. A new era within probiotics research has started with an increasing interest in the use of gut commensal bacteria as potential probiotics, such as strains belonging to the genera *Bacteroides, Clostridium, Bifidobacterium*, and *Faecalibacterium*, which predominate in the human gut microbiome (Langella et al., 2019).

At the same time, several studies dealing with health-promoting benefits associated with the consumption of fermented foods and beverages have been proposed. Global fermented foods—classified into nine major groups on the basis of raw materials—can be represented by more than 5,000 varieties being consumed around the world by billions of people. In the last 20 years, culture-independent methods have emerged as a convenient complement for analyzing the microbiota of fermented foods. Polymerase chain reaction-denaturing gradient gel electrophoresis (PCR-DGGE) was employed for monitoring microorganisms during food production, storage, and distribution (RT: “Molecular methods in food quality and safety” by Ercolini, 2011–2013). In the last decade, the high-throughput DNA sequencing (HTS) technologies have made possible the evaluation of microbiota of complex matrices, having a major influence on food microbiology for the determination of the whole genome sequence (WGS) of a single cultured isolate and for generating sequences of multiple microorganisms in a sample (metagenomics). The application of metagenomics can give information on the presence of spoilage and pathogen microorganisms or characterize unknown microbiota, particularly in fermented foods (Ronholm, 2018). A very high number of studies have been published in the “Food Microbiology” section on different traditional fermented foods and beverages. Aspects concerning the role of microbial consortia involved in the transformation of animal and raw plant materials in edible fermented foods with high nutritional value and that are rich in bioactive compounds beneficial to consumers were discussed in detail. The history of ethnic fermented foods and beverages dates back to more than 3,500 years ago and has evolved to preserve crops and dairies as fermented foods, often using back-slopping to inoculate the new batch by transferring an aliquot from the previous food batch and allowing for microbial adaptation and natural selection of strains. For this reason, the traditional, indigenous, or ethnic food fermentations represent a cultural heritage at a global level, harboring a huge genetic potential for undiscovered strains; research on this topic has to be improved through better exploration in the next years. At present, a very interesting research topic on “Microbiology of Ethnic Fermented Foods and Alcoholic Beverages of the World” has been proposed (Tamang et al., 2017). In these studies, Next Generation Sequencing (NGS) studies revealed new dimensions of microbial ecology.

The stress response in food microbes has been the focus of more than 400 articles published in the last 10 years, and more than 20 RTs have targeted microbial resistance. A wide range of food bacteria, pathogens or not, have been described to possess many adaptive mechanisms and specific stress responses that are useful to guarantee and improve fitness under specific environments. An important bacterial stress response is related to cross-protection, which plays a significant role in minimally processed foods. In fact, sub-lethal stress can induce multiple stress responses posing major public health concerns since many bacterial pathogens can become resistant to new preservation technology or processing. Many injured pathogens either retain or exhibit enhanced virulence in foods, thus making their detection crucial to safeguard the food supply chain. In addition, a cell fraction of the stressed bacterial population can remain metabolically active; they enter a non-culturable physiological state and represent a challenge for traditional food microbiology analytical methods. Future research should focus on the implementation of new methodologies for analytical methods able to detect and enumerate viable—but not culturable—cells as well as their stress responses and adaptation (Ruiz et al., 2017).

Bacterial pathogens associated with foodborne disease worldwide include *Salmonella enterica, Campylobacter jejuni, Escherichia coli, Listeria monocytogenes, Cronobacter sakazakii, Vibrio cholerae*, and *Vibrio parahaemolyticus*. In these last 10 years, 12 RTs have presented 293 articles related to foodborne pathogens. The subjects have ranged from foodborne pathogenic bacteria resurgences to pathogenesis and control strategies, antibiotic resistance, enteric virus, bacterial toxin, stress responses, applications of protective cultures, and bacteriocins for foods preservation, and new methods for the study foodborne pathogens. In general, many papers have focused on enteropathogenic bacteria (336), with specific studies being conducted on *Escherichia coli* (227), *Salmonella* spp. (167), *Listeria* spp. (153), *Campylobacter* spp. (104), *Vibrio* spp. (89), and *Yersinia* spp. (13).

Continuous monitoring of food contaminants and the identification of risk factors are crucial for assuring food safety. Many original research articles included in these RTs have addressed issues related to the genetic diversity, prevalence, resistance, and novel transmission vectors of pathogenic bacteria, but they have also reported new findings on bacterial pathogenesis, such as antimicrobial or desiccation resistance associated with diverse genotypes or the identification of virulence determinants produced and secreted by pathogenic bacteria. Among the future targets of food microbiology, it could be interesting to pursue new findings and studies on the expression of critical virulence factors, which allow for niche adaptation and successful colonization, such as the persistence in food processing facilities via growing predominantly as biofilms rather than in a planktonic mode (Jeanson and Thierry, 2015). New biological and non-biological innovation technologies, new compounds and treatment strategies, and advances in DNA sequencing technologies, with the characterization of bacterial genomes, have emerged for the control of foodborne pathogens; this must also be pursued further in the near future (Chen and Alali, 2018).

Several articles have focused on fermented foods, such as bread, cheese, wine, and others. Even if these foods have already been studied extensively in the past, the use of new technologies and omics approaches to implement the knowledge of how the microbiota affects quality and safety attributes of these foods and beverages has been encouraged, and this trend will be confirmed also in the future. For example, there are many fermented dairy products (in particular traditional ones) that have been poorly studied in terms of microbiological composition, microbial dynamics, and technological processes. These fermented foods represent a particular niche that could be rich with new positive and beneficial microbial strains influencing food quality and safety and that can also improve human health among other aspects.

Therefore, the microbiological integrity of the dairy food chain, the ecology of pathogenic and spoilage organisms, and the genomic analysis of these contaminants, such as novel strategies for their control, are important targets to be addressed. Nine RTs were proposed to discuss these objectives. However, other studies on different fermented foods following similar approaches have been published in the “Food Microbiology” section. For example, health and safety issues, particularly dealing with chemical and microbial potential hazards, have been related to fermented products of meat, vegetable, fish, rice, soybean, and corn origin. Among fermented beverages, seven RTs for a sustainable viticulture and winemaking, non-conventional yeasts and lactic acid bacteria (LAB) in winemaking, and the production of toxic compounds by microorganisms, such as ochratoxin and biogenic amines, were proposed. In general, the main relevant topics in the next years, related to fermented foods, can be summarized: microbiota involved in product fermentation; a selection of technological, protective, and probiotic starters as well as safety concerns related with their use; the genomic and metabolomics characterization of microorganisms with a technological impact on fermented products; the control and inhibition of pathogens and spoilage organisms; the relationship between technological procedures and microbiota of fermented foods; and traditional and ethnic fermented foods and beverages. In contrast to other habitats, foods are generally characterized by a not relevant number of microbial species. Among these, LAB play an essential role in the development of probiotics and starter cultures. In fact, LAB are an industrially important group of microorganisms used throughout the world for a large variety of food fermentations, such as those of dairy, wine, bread, vegetables, and others, much discussed in the RT “Industrial and health applications of LAB and their metabolites.” More than 30 RTs have focused on LAB, and more than 340 articles have been published on this topic. LAB in particular constitute a diverse group of Gram-positive, catalase-negative bacteria producing lactic acid. Some food-associated LAB obtained the status of “Qualified Presumption of Safety” (QPS) by the European Food Safety Agency (EFSA) or the “General Recognized As Safe” (GRAS) status by the U.S. Food and Drug Administration. Nowadays, the use of these microorganisms and their metabolites for food preservation has been extended to several additional bioactivities. The RT “Application of protective cultures and bacteriocins for food preservation” (Hammami et al., 2019) focused on antimicrobial substances produced by LAB inhibiting foodborne pathogens and spoilage microorganisms. These studies are in progress and will increase further in the near future, offering a promising alternative to chemical preservatives to ensure the quality and safety of ready-to-eat, extended-shelf-life, fresh-tasting, and minimally processed foods. Similar remarks can be made regarding *Saccharomyces* and non-*Saccharomyces* yeasts for alcoholic fermentation and *Bacillus* spp. for alkaline fermented foods. However, new starter cultures should be identified that involve natural adaptation and evolution, such as a direct selection of mutants with the desired properties, adaptative laboratory evolution, and genetic methods besides genome sequencing of wild type strains, for guiding safety assessments and strain-improvement activities (Johansen, 2018).

Looking forward, conventional food sources may be complemented by edible microbial biomass derived from bacteria, yeasts, filamentous fungi, or microalgae. Nowadays these groups of microorganisms are evaluated as an important and good source of proteins, vitamins, and beneficial bioactive compounds. It appears that the human population will increase up to about 9–12 billion people by the year 2100, and microorganisms could be an integral part of the sustainable production system. The “Food Microbiology” section of “Frontiers in Microbiology” could provide good guidance through its RTs on new advances in microbiology for the improved utilization, production, and supply of food in the food industry and related fields, which can help to ensure global food safety and security.

## Author Contributions

GS and AC gave the same contribution to the drafting of the article.

### Conflict of Interest

The authors declare that the research was conducted in the absence of any commercial or financial relationships that could be construed as a potential conflict of interest.
